# The Invasion Factor ODZ1 Is Upregulated through an Epidermal Growth Factor Receptor-Induced Pathway in Primary Glioblastoma Cells

**DOI:** 10.3390/cells13090766

**Published:** 2024-04-30

**Authors:** Carlos Velasquez, Olga Gutierrez, Maria Carcelen, Jose L. Fernandez-Luna

**Affiliations:** 1 Department of Neurosurgery, Hospital Universitario Marqués de Valdecilla, 39008 Santander, Spain; carlosjose.velasquez@scsalud.es; 2 Instituto de Investigación Marqués de Valdecilla (IDIVAL), 39008 Santander, Spain; molga.gutierrez@scsalud.es (O.G.); maria.carcelen93@gmail.com (M.C.); 3 Department of Anatomy and Cellular Biology, Universidad de Cantabria, 39011 Santander, Spain; 4 Department of Genetics, Hospital Universitario Marqués de Valdecilla, 39008 Santander, Spain; 5 Centro de Investigación en Red de Enfermedades Raras (CIBERER), 28029 Madrid, Spain

**Keywords:** ODZ1, EGFR, glioblastoma, migration, p38 MAPK

## Abstract

We have previously shown that the transmembrane protein ODZ1 promotes cytoskeletal remodeling of glioblastoma (GBM) cells and invasion of the surrounding parenchyma through the activation of a RhoA–ROCK pathway. We also described that GBM cells can control the expression of ODZ1 through transcriptional mechanisms triggered by the binding of IL-6 to its receptor and a hypoxic environment. Epidermal growth factor (EGF) plays a key role in the invasive capacity of GBM. However, the molecular mechanisms that enable tumor cells to acquire the morphological changes to migrate out from the tumor core have not been fully characterized. Here, we show that EGF is able to induce the expression of ODZ1 in primary GBM cells. We analyzed the levels of the EGF receptor (EGFR) in 20 GBM primary cell lines and found expression in 19 of them by flow cytometry. We selected two cell lines that do or do not express the EGFR and found that EGFR-expressing cells responded to the EGF ligand by increasing ODZ1 at the mRNA and protein levels. Moreover, blockade of EGF-EGFR binding by Cetuximab, inhibition of the p38 MAPK pathway, or Additionally, the siRNA-mediated knockdown of MAPK11 (p38β MAPK) reduced the induction of ODZ1 in response to EGF. Overall, we show that EGF may activate an EGFR-mediated signaling pathway through p38β MAPK, to upregulate the invasion factor ODZ1, which may initiate morphological changes for tumor cells to invade the surrounding parenchyma. These data identify a new candidate of the EGF–EGFR pathway for novel therapeutic approaches.

## 1. Introduction

Glioblastoma (GBM) is the most common malignant primary brain cancer and the invasion capacity is a key factor for its aggressiveness [1,2]. GBM cell migration and invasion are highly dynamic processes and involve, among others, the reorganization of the cytoskeleton [3,4]. We have shown that overexpression of the transmembrane protein ODZ1 in GBM primary cells increased the invasiveness of these cells by promoting cytoskeletal remodelling [3]. Furthermore, disruption of the ODZ1 protein by deleting the intracellular domain or blocking its expression significantly decreased the invasive capacity of tumor cells. ODZ1 has been related to different tumors through a chromosomal translocation involving Exon 2 of C11orf73 and Intron 19 of ODZ1. The fusion transcript is expected to trigger a nonsense-mediated decay mechanism resulting in mRNA degradation [5]. We identified a deletion of the entire ODZ1 gene in a patient with GBM [3] and cells from this patient had a deficiency in cytoskeletal organization. Thus, ODZ1-deficient cells are unable to form long actin-filled protrusions and have lower migration and invasion capacities. Invasiveness is a key hallmark of GBM causing tumor recurrence, generally in close proximity to the original tumor site [4]. However, targeting the invasive pathways of GBM, including cytoskeletal reorganization, cell migration, and degradation of the extracellular matrix, has not reached the stage of clinical development [4,6]. One way to improve the therapeutic intervention is to understand the interacting network between GBM cells and their microenvironment. ODZ1 can be upregulated by a number of pathways involved in GBM pathogenesis. Hypoxia is one of the main biological hallmarks of the GBM microenvironment and it is associated with higher levels of ODZ1 through epigenetic and HIF2α-mediated transcriptional mechanisms [7,8]. Moreover, IL-6 secreted by activated monocytes and the extracellular matrix protein fibronectin promote the expression of ODZ1 via activation of a Stat3-mediated transcriptional pathway [9]. This might be another relevant strategy for tumor cells to induce migration since immune cells, including activated monocytes/macrophages, comprise a significant amount of cells in GBM tumors. However, ODZ1 has not been linked to the epidermal growth factor receptor (EGFR), a key player in tumor malignancy in several cancer types, including GBM, and a target for therapeutic interventions [10]. A recent work described tumor regression in two patients with GBM treated with a CAR-T strategy engineered to target EGFRvIII, as well as wild-type EGFR [11]. Although the responses were transient, the study sheds light on the potential of this technology against main biomarkers in GBM and highlights the relevance of the EGFR as a key target. Other therapeutic strategies, including EGFR inhibitors, have not achieved the same degree of success seen in other cancers, partly due to the blood–brain barrier and intrinsic resistance [10,12]. Thus, deciphering potential paths forward to target the EGFR pathway may open new opportunities for therapies against GBM.

In this study, we showed that the invasion factor ODZ1 is upregulated by an EGFR–p38-MAP-kinase signaling pathway that might explain, at least in part, the higher migration capacity of GBM cells in the presence of EGF, which is highly reduced through the blockade of this pathway.

## 2. Materials and Methods

### 2.1. Cell Cultures

The IDH1/2 wild-type primary GBM cell lines used in this study were previously established from surgical specimens in our laboratory [13]. Tumor cells were maintained as neurospheres in serum-free DMEM/F12 medium (Invitrogen, Carlsbad, CA, USA) and plated at a density of 3 × 10^6^ live cells/60 mm plate. Neurospheres were dissociated every 4–5 days to facilitate cell growth. Cells were used between Passages 10 and 20. All cells were confirmed to retain their differentiation capacity, mainly towards astrocytes, reducing the stem cell markers CD133 and Sox2 and increasing the astrocytic marker GFAP, as described [13]. When indicated, GBM cells were incubated in the presence of 50 ng/mL EGF (Sigma-Aldrich, St. Louis, MO, USA), with or without 2 μg/mL Cetuximab (Merck KGaA, Darmstadt, Germany) or 10 μM SB203580 (Sigma-Aldrich, St. Louis, MO, USA).

All cells were tested for mycoplasma using the LookOut Mycoplasma qPCR Detection Kit (Sigma-Aldrich) within one week before the experimental work.

When indicated, cells were cultured to create a confluent monolayer and then a wound was generated by manually scraping the monolayer with a 23 G needle (0.60 × 25 mm). The cell culture was incubated with EGF and images were taken at different time points.

### 2.2. Analysis of RNA-Seq Data from TCGA Database

We analyzed the ODZ1 gene expression according to the EGFR mutational status in 214 GBM samples by using RNA-seq data from The Cancer Genome Atlas (TCGA) database following a protocol previously reported [14]. All single nucleotide mutations and insertions/deletions (indels) of the EGFR gene were included.

### 2.3. Gene Expression Analyses

The expression of individual genes was evaluated by qPCR on total cellular RNA, as previously described [3]. Additionally, cDNA was generated and amplified using the following primers: β-Actin (5′GCGGGAAATCGTGCGTGACATT and 5′GATGGAGTTGAAGGTAGTTTCGTG), ODZ1 (5′ACTCAAGAGATGGAATTCTGTG and 5′CTTAGTGCATGGTCAGGTG), MAPK14 (5′CCAGGGGCTGAGCTTTTGAA and 5′TCGGCCACTGGTTCATCATC), MAPK11 (5′AGGAGCTGAACAAGACCGTG and 5′ACACTTCGCTGAAGTCCTCG). Additionally, qPCR was performed in a QuantStudio 5 real-time PCR system (Life Technologies, Carlsbad, CA, USA).

### 2.4. PCR-Based Discrimination of EGFR Variants

The deletion of Exons 2–7 of the EGFR that characterizes the EGFRvIII mutant variant of the receptor was analyzed by cDNA amplification with primers in Exon 1 (5′GAGTCGGGCTCTGGAGGAAA) and Exon 8 (5′CCATCTCATAGCTGTCGGCC), which amplify fragments of 893 bp (wild-type EGFR) and 92 bp (EGFRvIII). Fragments were detected by agarose gel electrophoresis after 35 cycles of amplification with an annealing temperature of 60 °C.

### 2.5. Flow Cytometry

Cells collected from culture flasks were washed with phosphate buffer saline containing 3% bovine serum albumin (Sigma-Aldrich) and then incubated with an anti-EGFR antibody labeled with Alexa Fluor 488 (AY13, Biolegend, San Diego, CA, USA). Stained cells were analyzed in a FACS Canto II flow cytometer (BD Biosciences, La Jolla, CA, USA).

### 2.6. Western Blot Analysis

Total proteins from GBM cells were separated on 8% polyacrylamide gels and transferred to nitrocellulose. Blots were incubated with antibodies against pMAPKAPK2-Thr222 (9A7, Cell Signaling, Danvers, MA, USA), MAPKAPK2 (D1E11, Cell Signaling), p38α/β MAPK (Y122, Abcam, Cambridge, UK), ODZ1 (AF6324, R&D Systems, Minneapolis, MN, USA), and αTubulin (B-5-1-2, Santa Cruz Biotechnology. Santa Cruz, CA, USA), followed by secondary anti-rabbit, anti-sheep, or anti-mouse antibodies conjugated to horseradish peroxidase (Santa Cruz Biotechnology). When indicated, protein bands were quantitated by using ImageJ software (v1.53k, Windows version of NIH Image, https://imagej.net/ij/, accessed on 5 November 2023).

### 2.7. Detection of EGFR Gene Amplification

EGFR amplification was determined by using the SALSA MLPA Probemix P135 (MRC Holland, Amsterdam, The Netherlands), following the manufacturer’s instructions. This Probemix includes 30 MLPA probes for the EGFR gene, covering all EGFR exons except Exon 11. The resulting fragments were separated by capillary electrophoresis using a SeqStudio analyzer (Applied Biosystems, Waltham, MA, USA).

### 2.8. Gene Silencing

For MAPK11/14 knockdown experiments, we used 3 unique 27mer siRNA duplexes specific for either MAPK11 (Locus ID 5600) or MAPK14 (Locus ID 1432) and scrambled negative control siRNA duplexes (all from Origene, Rockville, MD, USA). Cells were transfected with 50 pmol siRNAs by using Lipofectamine RNAiMAX (Invitrogen) and 48 h later, protein expression was analyzed by Western Blotting.

### 2.9. Statistical Analysis

All statistics were calculated with the SPSS statistical package (version 13.0). Data are presented as the mean ± SD of three independent experiments. Differences between groups were tested for statistical significance using the unpaired 2-tailed Student’s *t*-test. The significance level was set at *p* < 0.05.

## 3. Results

### 3.1. Activation of EGFR Correlates with the Expression of ODZ1

**Figure 1:**
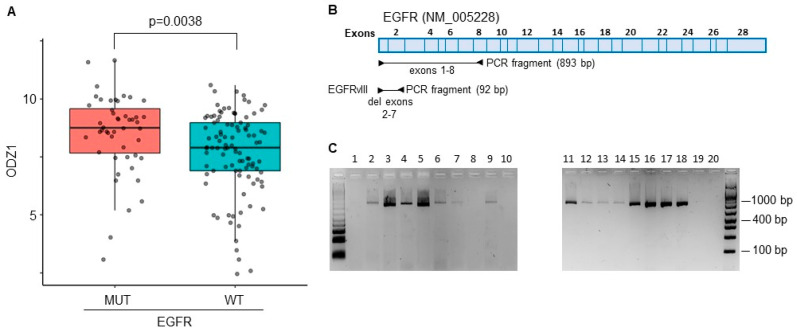
The constitutively active mutant EGFRvIII correlates with higher levels of ODZ1. (**A**) ODZ1 expression was identified from RNA-seq data in TCGA datasets that provided information on a GBM cohort containing 214 patient samples carrying either the wild-type EGFR or the mutant variant EGFRvIII. (**B**) Schematic representation of the EGFR gene showing the PCR design to discriminate the EGFR from EGFRvIII. (**C**) PCR analysis of 20 primary GBM cell lines using the experimental design described in (**B**).

The constitutively active mutant EGFRvIII is one of the most commonly detected gene variants in GBM [15]. A correlation between EGFRvIII and ODZ1 expression was identified from RNA-seq data in TCGA datasets that provided information on a GBM cohort containing 214 patient samples. Patients with GBM cells carrying the mutant version of the EGFR expressed higher levels of ODZ1 mRNA than those with the wild-type EGFR (Figure 1A).

**Figure 2:**
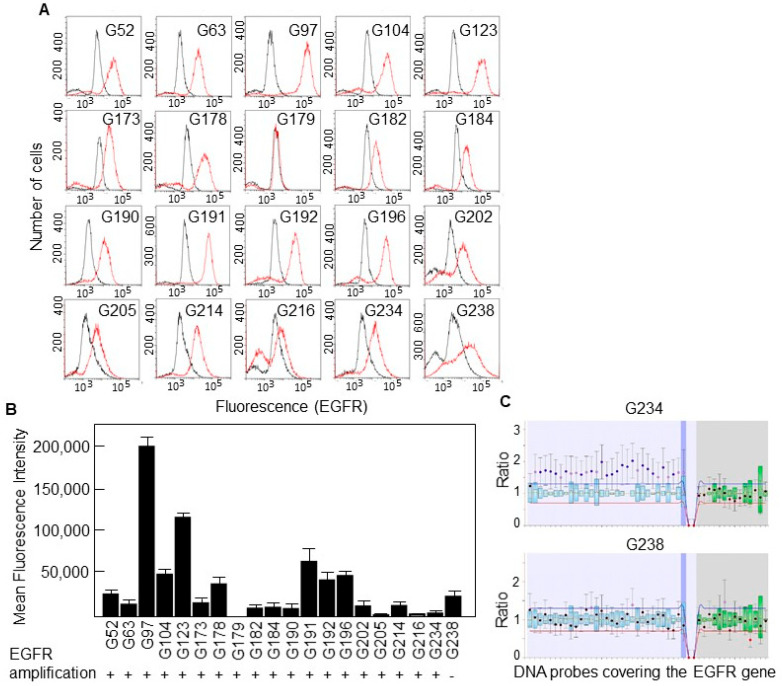
Expression of the EGFR protein in primary GBM cells. (**A**) EGFR expression was determined by FACS analysis. Red line, anti-EGFR antibody. Black line, isotype-matched control antibody. (**B**) Mean fluorescence intensity of FACS results and the gene amplification status of the EGFR in all the cell lines. Cell lines are ordered in the same way as in Figure 1C. (**C**) Representative result of a MLPA assay showing amplification of the entire EGFR gene.

We analyzed the presence of EGFRvIII in 20 different GBM cell cultures derived from surgical specimens by using a PCR approach, with primers flanking Exons 2–7 (Figure 1B). The deleted variant renders a PCR fragment of 92 bp while, with the reference allele, we observe a fragment of 893 bp. We could not detect the presence of the deleted variant in any of the cell lines (Figure 1C), not even following overamplification with a high number of PCR cycles. Then, we studied the expression of the EGFR protein in all 20 cell lines by flow cytometry and found that virtually all of them expressed the receptor; although, the levels were significantly different, from almost no expression in G179 to the highly positive G97 cells (Figure 2A,B).

EGFR amplification has been identified as a genetic hallmark of primary GBM and occurs in approximately 40–60% of these cases [16]. We performed MLPA analyses to detect copy number variations of the EGFR gene in all cell lines and found that all of them, but one, carried an amplified variant of the gene (Figure 2B,C).

### 3.2. ODZ1 Is Upregulated through an EGF–p38 Pathway

**Figure 3:**
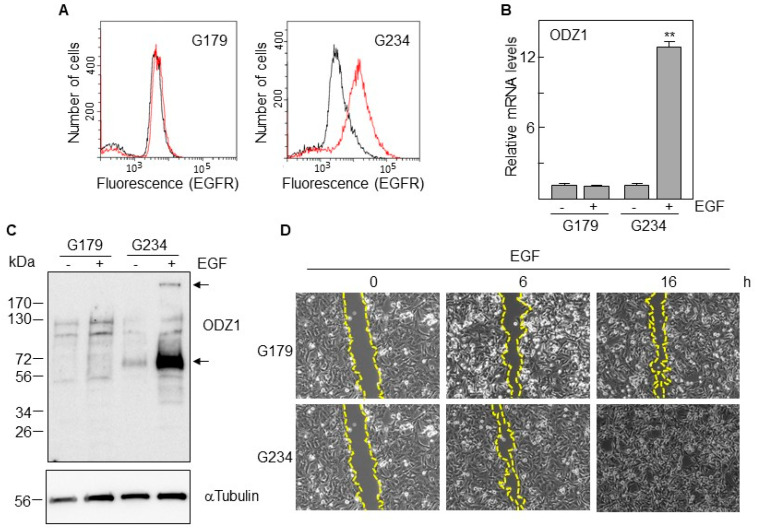
EGF induces the expression of ODZ1. (**A**) FACS analysis of two GBM cell lines expressing, or not, EGFR (same as in Figure 2A). (**B**) GBM cells were incubated with EGF and the mRNA levels of ODZ1 were determined after 24 h by qPCR. Histograms represent the mean of three independent experiments ± S.D. Asterisks represent significant differences (** *p* < 0.01). (**C**) Western Blot analysis confirmed that the exposure of GBM cells to EGF also promoted the expression of ODZ1 at the protein level. Additionally, αTubulin was used to ensure equal loading. Arrows indicate the native high-molecular-weight ODZ1 protein and its most frequent proteolytic fragment of 70 kDa. (**D**) Cells were incubated in the presence of EGF and migration was determined at different time points by using an in vitro wound healing assay.

We selected two cell lines with marked differences in the expression of EGFR, G179, and G234 (Figure 3A). The first one, which has EGFR levels almost undetectable by flow cytometry, did not increase the expression of ODZ1 mRNA following culture with EGF. However, EGFR-expressing G234 cells increased, about 12-fold, the levels of ODZ1 in response to EGF (Figure 3B).

This result was confirmed when we analyzed the ODZ1 protein. Although the native protein is about 300 kDa, smaller fragments, mostly that of 70 kDa, are frequently detected in Western Blot analyses due to partial proteolysis [3]. Consistent with the mRNA data, the ODZ1 protein was not elevated in G179 cells following treatment with EGF; however, the G234 cell line showed a clear upregulation of ODZ1 in response to EGF and both the high-molecular-weight native protein and the 70 kDa fragment could be detected (Figure 3C). In line with the known migratory activity of ODZ1, G179 cells expressing no or very low levels of the ODZ1 protein closed the scratched area slower than ODZ1-expressing G234 cells in response to EGF, as assessed by means of a wound-healing assay (Figure 3D). After 16 h of treatment, G234 cells completely closed the scratched area; whereas, an empty area still remained with G179 cells.

**Figure 4:**
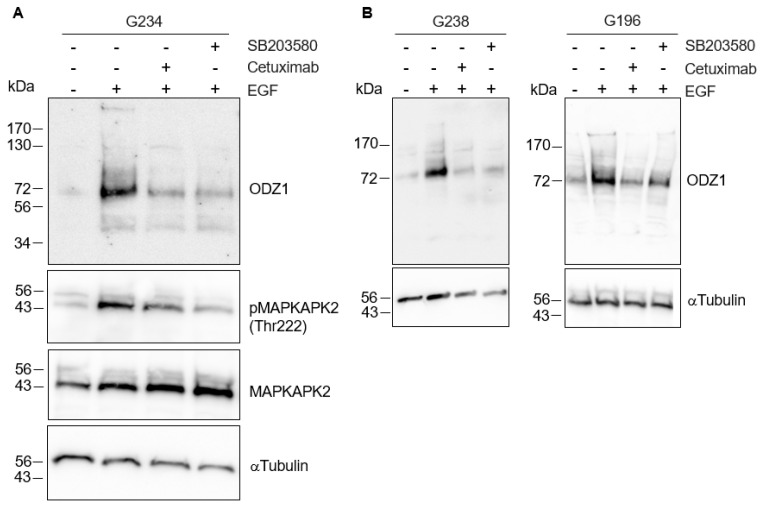
The blockade of the EGFR–p38 signaling downregulates the levels of ODZ1. (**A**) GBM cells were treated with EGF in the absence or in the presence of the EGFR-blocking antibody Cetuximab or the specific p38 MAPK inhibitor SB203580. Western Blot analysis confirmed that the blockade of EGFR/p38 inhibited the phosphorylation of MAPKAPK2 at Thr222, a target of p38, and reduced the expression of the ODZ1 protein. (**B**) Downregulation of ODZ1 following inhibition of EGFR/p38 was confirmed in two additional GBM cell lines. MAPKAPK2 and αTubulin were used to ensure equal loading.

As expected, when G234 cells were treated with EGF in the presence of the EGFR-blocking antibody Cetuximab, the EGF-mediated upregulation of ODZ1 was abolished or highly reduced (Figure 4). The EGF binding to its cognate receptor is able to activate different signaling pathways, including p38 MAPK. It has been shown that in glioma cells, activation of p38 contributes to tumor invasion and is correlated to tumor grade, being considered a potential oncogenic factor in brain tumors [17]. Thus, we asked whether this pathway could mediate the overexpression of ODZ1 induced by the EGF–EGFR binding complex. Incubation of G234 cells with SB203580, a specific p38 MAPK inhibitor that blocks its catalytic activity [18], drastically reduced both the levels of ODZ1 and the phosphorylation of MAPK-activated protein kinase 2 (MAPKAPK2), a direct target substrate of p38. A similar result, although to a lesser extent, was obtained following treatment with Cetuximab (Figure 4A). Downregulation of ODZ1 protein levels in GBM cells following the inhibition of EGFR/p38 was confirmed by analyzing the other two cell lines, G238 and G196, from the collection described in Figure 2 (Figure 4B).

**Figure 5:**
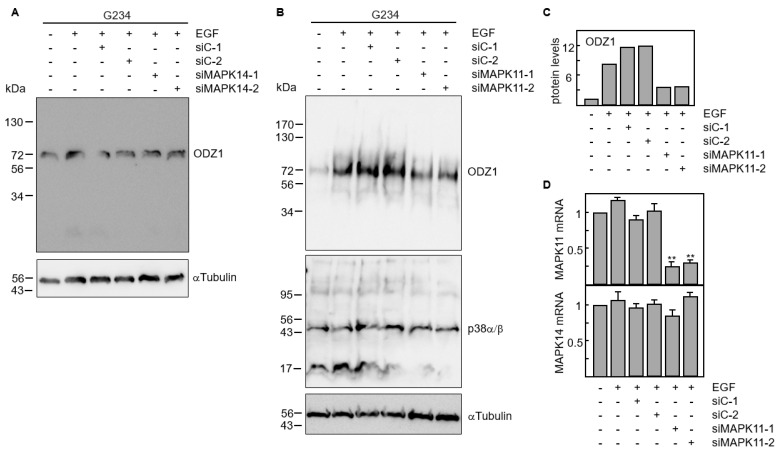
RNA interference by p38-specific siRNAs neutralizes the EGF-promoted upregulation of ODZ1. (**A**) GBM cells were transfected with two MAPK14-specific siRNAs or two control siRNAs. Following incubation with EGF, the protein levels of ODZ1 were determined by Western Blot analysis. (**B**) Same experimental design as in A but uses MAPK11-specific siRNAs. Additionally, αTubulin was used to ensure equal loading. (**C**) Quantification of ODZ1 protein levels of the experiment shown in B by using imageJ software (v1.53k). (**D**) Cells carrying siRNAs specific to MAPK11 were analyzed for the expression of either MAPK11 or MAPK14 mRNA by qPCR. Histograms represent the mean of three independent experiments ± S.D. ** *p* < 0.01.

The results obtained with chemical and biological inhibitors were confirmed by knocking down the gene expression of p38 MAPK with siRNAs. Four p38 isoforms have been described, including p38α (coded by MAPK14 gene) and p38β (coded by MAPK11 gene), which are ubiquitously expressed and share a high sequence identity; whereas, the other two isoforms have a more limited tissue-specific expression [19]. We knocked down either MAPK11 or MAPK14 using two specific siRNAs. Downregulation of MAPK14 did not modify the ODZ1 protein levels (Figure 5A). However, transfection of GBM cells with MAPK11-specific siRNAs significantly reduced (about four-fold) the ODZ1 protein expression (Figure 5B,C). As expected, these siRNAs downregulated MAPK11 but not MAPK14 (Figure 5D). This might be the reason why we do not observe a clear reduction in the protein levels of p38 as the antibody recognizes both α and β isoforms.

## 4. Discussion

We have previously described that ODZ1 is an important factor for GBM cells to invade the surrounding tissue [3]. This invasion factor is upregulated when GBM cells are cultured under hypoxic conditions [7] and it also responds to microenvironmental stimuli, such as IL-6, which is released by infiltrating macrophages, and fibronectin, a major component of the extracellular matrix [9]. Thus, GBM cells have the potential to use different microenvironmental elements to promote the expression of an invasion factor that has been demonstrated to provide a major contribution to the spreading of tumor cells, both in vitro and in vivo [3]. Ligand-dependent/independent activation of the EGFR is present in about 60% of primary GBM cells [20]. Of note, spatial distribution analysis showed that EGFR overexpression was localized in the infiltrating tumor edge [21], providing further evidence of the relevance of the EGFR for the invasion capacity of GBM cells. Based on this, we asked whether the EGFR could promote the expression of ODZ1. Although none of the 20 primary GBM cell lines tested carried the constitutively active EGFRvIII mutant, EGFR gene amplification, a hallmark genetic aberration in GBMs, occurred in almost all of them, expressing varying levels of the EGFR. Aberrant EGFR signaling mechanisms, including the overexpression of ligands and receptors, EGFR gene amplification, and activating mutations, have been shown in a number of cancers, such as non-small-cell lung cancer, colorectal carcinoma, breast cancer, and head and neck squamous cell carcinoma [22]. We have shown that GBM cells are able to induce ODZ1 expression at the mRNA and protein levels in response to EGF, pointing to the existence of an EGFR–ODZ1 transcriptional pathway. The EGFR has been shown to promote tumor cell migration/invasion through different transcriptional mechanisms that include the STAT-mediated upregulation of metalloproteinase-1 [23], expression of the actin-binding protein Fascin [24], and expression of invasion-associated genes through the activation of AP-1 transcription factors [25]. In GBM cells, the activation of p38 MAPK is associated with tumor invasion and correlates with tumor grade, which indicates that this kinase could be an oncogenic factor that contributes to brain tumor progression [17]. In line with this, we found a positive correlation between the levels of ODZ1 and activation of p38 MAPK. The p38 MAP kinases are encoded by four genes: MAPK14 (p38α), MAPK11 (p38β), MAPK12 (p38γ), and MAPK13 (p38δ). MAPK14 and MAPK11 are highly homologous and co-located on Chromosome 22. Additionally, p38α and p38β are expressed in most cell types of the brain, including astrocytes and microglia [26,27]. Thus, we studied their role in mediating the expression of ODZ1 by using MAPK14/MAPK11-specific siRNAs and found a correlation between ODZ1 and MAPK11 but not MAPK14. As mentioned above, both p38α and p38β share high sequence homology, about 80% [28], which might explain why MAPK11 siRNAs downregulate the mRNA but the Western Blot analysis did not show a clear reduction in the p38β protein as the antibody used recognizes both isoforms. MAPK11 (p38β) has been shown to promote the expression of lipocalin 2, which is correlated with increased invasion and metastases in different types of tumors [29]. Furthermore, MAPK11 plays a key role in the invasion and migration of cancer cells in breast, endometrial, and hepatocellular carcinomas [29,30]. The implication of the EGFR–p38 MAPK axis in cell migration has been extensively described [17,31,32]. These results are consistent with previous data showing that the downregulation of ODZ1 by small interfering RNAs greatly impeded the migration and invasion of GBM cells, both in vitro and in vivo [3,7,9].

## 5. Conclusions

**Figure 6:**
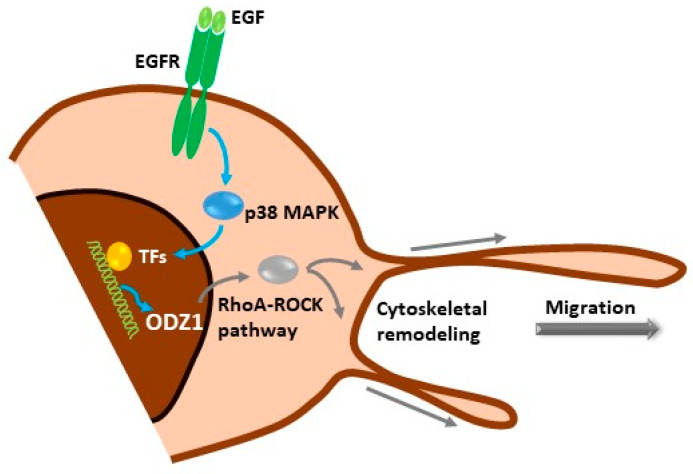
Schematic representation of the EGFR–p38–ODZ1 transcriptional pathway. EGF, present in the tumor microenvironment, binds to its receptor and triggers the activation of p38 MAPK, which induces a transcriptional mechanism to upregulate ODZ1, which is a known migration factor in GBM cells through the RhoA–ROCK pathway.

Our data show a novel signalling mechanism, initiated by activation of the EGF receptor in GBM cells, which promotes the p38-MAPK-dependent expression of ODZ1, a previously characterized invasion factor for tumor cells. ODZ1 is able to reorganize the cytoskeletal network and promote tumor cell migration (Figure 6). Overall, we have been able to connect one of the biochemical hallmarks of GBM, overexpression of EGFR, with ODZ1, a key player in promoting the invasion of GBM cells.

## Data Availability

Data available on request.
